# Estimating effective survey duration in camera trap distance sampling surveys

**DOI:** 10.1002/ece3.10599

**Published:** 2023-10-13

**Authors:** Hjalmar S. Kühl, Stephen T. Buckland, Maik Henrich, Eric Howe, Marco Heurich

**Affiliations:** ^1^ Senckenberg Museum for Natural History Görlitz Senckenberg – Member of the Leibniz Association Görlitz Germany; ^2^ International Institute Zittau, Technische Universität Dresden Zittau Germany; ^3^ German Centre for Integrative Biodiversity Research (iDiv) Halle‐Jena‐Leipzig Leipzig Germany; ^4^ Centre for Research into Ecological and Environmental Modelling University of St Andrews, The Observatory St Andrews UK; ^5^ Department of National Park Monitoring and Animal Management Bavarian Forest National Park Grafenau Germany; ^6^ Faculty of Environment and Natural Resources Albert Ludwigs University of Freiburg Freiburg Germany; ^7^ Wildlife Research and Monitoring Section Ontario Ministry of Natural Resources and Forestry Peterborough Ontario Canada; ^8^ Institute for Forest and Wildlife Management Inland Norway University of Applied Science Koppang Norway

**Keywords:** animal abundance, camera recovery time, camera trap distance sampling, retrigger delay, still images, video

## Abstract

Among other approaches, camera trap distance sampling (CTDS) is used to estimate animal abundance from unmarked populations. It was formulated for videos and observation distances are measured at predetermined ‘snapshot moments’. Surveys recording still images with passive infrared motion sensors suffer from frequent periods where animals are not photographed, either because of technical delays before the camera can be triggered again (i.e. ‘camera recovery time’) or because they remain stationary and do not immediately retrigger the camera following camera recovery time (i.e. ‘retrigger delays’). These effects need to be considered when calculating temporal survey effort to avoid downwardly biased abundance estimates. Here, we extend the CTDS model for passive infrared motion sensor recording of single images or short photo series. We propose estimating ‘mean time intervals between triggers’ as combined mean camera recovery time and mean retrigger delays from the time interval distribution of pairs of consecutive pictures, using a Gamma and Exponential function, respectively. We apply the approach to survey data on red deer, roe deer and wild boar. Mean time intervals between triggers were very similar when estimated empirically and when derived from the model‐based approach. Depending on truncation times (i.e. the time interval between consecutive pictures beyond which data are discarded) and species, we estimated mean time intervals between retriggers between 8.28 and 15.05 s. Using a predefined snapshot interval, not accounting for these intervals, would lead to underestimated density by up to 96% due to overestimated temporal survey effort. The proposed approach is applicable to any taxa surveyed with camera traps. As programming of cameras to record still images is often preferred over video recording due to reduced consumption of energy and memory, we expect this approach to find broad application, also for other camera trap methods than CTDS.

## Graphical abstract

Camera trap distance sampling (CTDS) has been previously formulated for recording of videos. Here, we extend CTDS methodology to trigger‐based recording of still images, which is the most common approach in wildlife surveys.

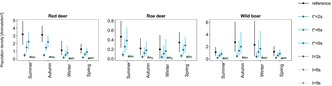

## 1 INTRODUCTION

With ongoing loss of biodiversity and decline of wildlife populations, species monitoring has become a major activity in applied conservation and research (e.g. Moussy et al., 2022; Nichols & Williams, 2006). Animal abundance in particular is a key parameter in ecological processes and essential for the assessment of species conservation status (Burton et al., 2015). Remote camera trapping has become a widely practised approach for assessing species occurrence, community composition, density and abundance (e.g. Bessone et al., 2020; Corlatti et al., 2020; Nichols & Karanth, 2011; Rowcliffe et al., 2008). Camera traps can be left in the field for several months and are activated either at regular intervals (time‐lapse photography) or by temperature differences and motion (e.g. a mammal or bird with a surface temperature higher than ambient temperature) via a passive infrared (PIR) motion sensor (Welbourne et al., 2016). A photograph, photo series or video is recorded following activation.

The first studies to estimate animal abundance from camera trapping data used capture–recapture methods (e.g. Karanth, 1995; Noss et al., 2012) which require that individual animals can be identified (commonly referred to as ‘marked population’ approaches). With camera traps, however, marked population approaches are only applicable to species with unique and recognisable physical characteristics, such as pelage patterns. For estimating abundance of ‘unmarked populations’, that is when individuals cannot be distinguished easily, several methods have been developed in recent years (Campos‐Candela et al., 2018; Chandler & Royle, 2013; Howe et al., 2017; Moeller et al., 2018; Nakashima et al., 2018; Rowcliffe et al., 2008). Spatially explicit models for unmarked animals (‘spatial count’ models) require spatially intensive sampling to detect the same animals at more than one location and yield imprecise estimates in the absence of additional data to inform the scale of individuals' movements, but do not require that cameras are programmed to record videos or on time‐lapse (Chandler & Royle, 2013). The random encounter model (REM, Jourdain et al., 2020; Rowcliffe et al., 2008) and time‐to‐event model (TTE) (Moeller et al., 2018) can also work with single sensor‐triggered photographs as long as an independent, reliable estimate of animal movement speed is available; it is often necessary to estimate movement speed directly from the camera trap data to avoid bias (Palencia et al., 2022; Rowcliffe et al., 2016). Other methods such as Moeller et al.'s (2018) space‐to‐event (STE) and instantaneous sampling (IS) estimators use time‐lapse photography to circumvent the requirement to account for animal movement. Nakashima et al.'s (2018) Random Encounter and Staying Time (REST) model and Campos‐Candela et al.'s (2018) home‐range based estimator also requires video surveys. Camera trap distance sampling (CTDS) avoids the need to estimate speed of movement or staying time by discretizing the survey duration into instantaneous ‘snapshot moments’ *t* units of time apart and calculating temporal survey effort as the survey duration divided by *t* (Howe et al., 2017). Howe et al. (2017) recommended programming cameras to record video when triggered to ensure distances could be measured at these predefined moments, and this formulation is also well‐suited for high‐frequency time‐lapse photography. However, CTDS has since also been applied in camera trap surveys with single images (e.g. Corlatti et al., 2020; Harris et al., 2020).

The performance of both REM and CTDS have been assessed in a number of field studies (e.g. Bessone et al., 2020; Cappelle et al., 2021; Cusack et al., 2015; Kavčić et al., 2021; Mason et al., 2022; Pal et al., 2021; Palencia et al., 2021) and have been validated with populations of known size (e.g. Cappelle et al., 2019; Harris et al., 2020; Rowcliffe et al., 2008). Other methods have received little testing with real data (but see Garland et al., 2020; Nakashima et al., 2020; Palencia et al., 2021 for the REST model, and Loonam et al., 2021 for TTE and STE). Several studies have compared subsets of these methods in terms of their assumptions, robustness to violations of assumptions, ease of implementation and their ability to produce accurate and precise results for different species and under different sampling scenarios; these studies did not recommend a particular model for use in all situations (Gilbert et al., 2021; Palencia et al., 2021; Santini et al., 2022).

Unfortunately, recording video or high‐frequency time‐lapse requires more energy and memory and thus more visits to camera locations during a survey. Time‐lapse surveys with long intervals between pictures may yield sparse data and fail to detect rare species. Given constraints on power supply and memory most camera trap surveys rely on PIR motion sensors and record single images or short bursts (≤1 s) of images rather than recording long bursts (over several seconds) or videos which require more memory and consume more power. Such surveys may not always yield data that conform to the assumptions of the statistical method underlying CTDS, which may result in biased estimates of density, but see, for example Corlatti et al. (2020) and Harris et al. (2020) for estimating animal abundance using CTDS and image‐based recording.

If technological limitations or animal behaviour prevent us from detecting animals at predefined snapshot moments, that we expect to be detected with a high probability based on their location relative to the camera, estimates of abundance will be negatively biased. For example, it has already been established that estimators should be corrected to account for periods of time when animals are not available for detection by camera traps because they are immobile (during long periods of sleep or rest) or because they are outside the vertical range of camera traps (Cappelle et al., 2019; Corlatti et al., 2020; Howe et al., 2017; Palencia et al., 2022). It has also been acknowledged that slow trigger speeds can cause missed detections of animals that pass quickly through the narrow part of the sector monitored at short distances from cameras (Corlatti et al., 2020; Howe et al., 2017). However, PIR motion sensor‐based recording of pictures requires consideration of another yet neglected issue: short (<20 s on average) intervals between consecutive pictures that prevent animals which remain for several seconds within the field of view (FOV) of the camera from being redetected. There are two causes of such delays: (1) Camera traps frequently have technological recovery times of several seconds after a picture has been taken even though manufacturers may specify shorter (<2 s) recovery times (Corlatti et al., 2020), and (2) animals that remain stationary in the FOV may not retrigger cameras continuously. Recovery times likely vary among models, and may also vary with, for example ambient temperature, humidity, image resolution, writing speed and state of memory cards and batteries. The second reason for the time difference between consecutive images, which we refer to as ‘retrigger delays’ is at least partly a function of animal movement behaviour, and therefore likely to be species‐, population‐ or group‐specific. In principle, bias induced by longer‐than‐expected recovery times and retrigger delays applies to PIR motion sensor‐based recording of both pictures and video. However, where videos or bursts are long relative to the time between records of the same animal within a passage through the FOV, the effect should become small or even negligible; this is not the case where only single images or short bursts are recorded each time the sensor is triggered.

Both camera recovery time and retrigger delay influence effective survey effort. When using CTDS, cameras will not record during all predefined snapshot moments when animals are present in the FOV due to these two effects. If not taken into account, temporal survey effort will be overestimated. Consequently, the temporal effort term (*T*/*t*) that expresses the number of snapshot moments during a survey as defined by Howe et al., 2017 requires adaption. *t* can no longer be defined as the time interval between predefined snapshot moments but needs to be redefined as the ‘mean time interval between triggers’ to take the extended time between consecutive camera trap images into account. Previous studies have suggested to derive *t* from experimentally tested camera recovery time, that is by human movement in front of a set of cameras and defining *t* as the minimum time interval between triggers (e.g. Corlatti et al., 2020; Harris et al., 2020).

Here, we propose a different approach to avoid negative bias in CTDS estimates of animal abundance when cameras are programmed to record single images or short bursts following the triggering of a PIR motion sensor, as opposed to long bursts or videos when triggered, or on time‐lapse mode. Rather than simply selecting the time interval between predetermined snapshot moments when distances to animals are determined (parameter *t* in the CTDS formula for estimating density; Equations (2), (3), (4) below), as recommended by Howe et al. (2017) when recording video, we estimate (t) as a function of mean camera recovery time and mean retrigger delay. We apply the approach to survey data on wild boar (*Sus scrofa*), red (*Cervus elaphus*) and roe deer (*Capreolus capreolus*) and show that careful truncation of interval data is critical to avoid contamination of the time interval distribution of the same animals by detections of different animals or animal groups arriving in a camera's FOV that are not relevant. We show that negative bias in estimated abundance can be large, if camera recovery times and retrigger delays are not accounted for.

## 2 MATERIALS AND METHODS

### 2.1 The point transect model

In conventional point transect distance sampling, a human observer makes observations in all directions from the centre of the transect. Radial distances to observed animals are recorded and used to estimate detection probability (*p*). The estimator of animal density is

(1) $$\;\hat{D}=\frac{\sum_{k=1}^{K}n_{k}}{\mathrm{k\pi}w^{2}\;\hat{p}}$$

where *K* is the number of point transects, $n_{k}$ is the number of observations on point transect *k*, *w* is the truncation distance beyond which animal observation distances are discarded, $\hat{p}$ is the estimated detection probability within *w* (Buckland et al., 2001). This conventional point transect model has been extended to accommodate to the use of camera traps.

### 2.2 The CTDS model

The CTDS model is simply a modified point transect distance sampling model:

(2) $$\hat{D}=\frac{\sum_{k=1}^{K}n_{k}}{\mathrm{\pi w}²\sum_{k=1}^{K}e_{k}{\hat{P}}_{k}}\frac{1}{\hat{A}},$$

where *K* is the number of camera trap locations, $n_{k}$ is the number of animal observations at camera location *k*, *w* is the truncation distance beyond which animal observation distances are discarded, ${\hat{P}}_{k}$ is the estimated detection probability within w at location *k*, and $e_{k}$ is sampling effort at location *k* (for conventional distance sampling with human observers $e_{k}$ is simply the number of visits to the point; Buckland et al., 2001, 2004). The estimate of the proportion of time spent active per day $\hat{A}$ is required to account for periods when animals are not available for detection. For CTDS the effort term is redefined to include two major differences. First, a camera trap does not cover a full circle, as is the case with human observers in conventional point transect distance sampling. Instead, a camera trap covers only a fraction, frequently with an opening angle between 30 and 50°. Second, the number of visits by a human in conventional point transect distance sampling is replaced by ‘snapshot moments’. Snapshot moments are the times when animal observations and their distances from the camera are recorded. More specifically, a 1‐min video clip recorded by a camera trap with 20–30 frames per second has about 1200–1800 frames. Not all of these are taken for analyses, as this would be too time‐consuming and would provide no additional information compared to a reduced data set of, for example one observation every 2–3 s. Howe et al. (2017) formulated the CTDS model to accommodate data from camera traps by redefining the effort term as:

(3) $$e_{k}=\frac{\theta T_{k}}{2\mathrm{\pi t}},$$

where *θ* is the horizontal angle of view of the camera in radians (such that $\frac{\theta }{2\pi }$ describes the fraction of a circle monitored), $T_{k}$ is the duration a camera trap is deployed at location *k*, and *t* is the time interval between predefined snapshot moments when camera‐to‐animal distances are measured, such that $\frac{T_{k}}{t}$ quantifies the number of opportunities in time to detect animals in the FOVs of cameras. Substituting this formula for $e_{k}$ into the above equation yields:

(4) $$\hat{D}=\frac{2t\sum_{k=1}^{K}n_{k}}{\theta \omega^{2}\sum_{k=1}^{K}T_{k}{\hat{P}}_{k}}\frac{1}{\hat{A}}$$

For PIR motion sensor‐based video recording and time‐lapse photography, *t* is known and at the discretion of researchers; Howe et al. (2017) suggested setting *t* to 0.25–3 s when recording video to avoid positive bias in observed distances for large values of *t* and to balance sample size and precision versus data processing effort. In order to represent animal movement well, *t* needs to be small enough to record representative positions of the animal path throughout a passage through the FOV (Howe et al., 2017). It is important to note that *t* defines the interval between predefined snapshot moments independent of animal observations. More specifically, the onset of snapshot moments is not defined based on, for example the first observation, but as specific times of day. When cameras are programmed to record single images or short bursts when triggered, *t* cannot be defined as proposed by Howe et al. (2017). One reason is that many observations would not predefined snapshot moments, but in‐between. A second complication with trigger‐based recording of still images is that intervals are highly variable when cameras record animals, depending on camera hardware and movement of animals, due to unknown and variable camera recovery times and retrigger delays. To address these issues previous studies have used, for example the minimum interval between retriggers tested in experimental setups to derive a value for *t* (e.g. Corlatti et al., 2020; Harris et al., 2020). Here, we propose to estimate this parameter and thus the realised temporal effort of the CTDS survey, from combined estimates of mean camera recovery time and mean retrigger delays derived from time interval distributions of consecutive pictures.

### 2.3 Estimating *t*

A direct approach to estimating *t* would be to examine consecutive images, determine whether successive detections are of the same individual and simply take the sample mean of the intervals between successive detections. However, this requires a lot of time and effort to track an individual within one passage through the field of view and to record the intervals within such a series. By contrast, a statistical approach to derive non‐observation times (hereby referred to as ‘mean time intervals between triggers’) from time interval distribution data gives results very quickly. As time interval data are a mixture of times between consecutive pictures of the passage of the same animal through the FOV (mostly short intervals) and detections of other individuals (includes longer intervals), we first need to truncate our data. This is meant to remove the longer time intervals that mainly stem from images of different animals entering the FOV at different times and gaps that can be explained by an animal not being visible, for example behind vegetation, which do not need to be considered here.

We truncate times between successive triggers of the camera at *T* so that we only analyse time intervals *t* for which t≤T. We can write

(5) t=r+v

where *r* is the camera recovery time after triggering, *v* is the time until the animal retriggers the camera after recovery, and *T* denotes truncation time and is not related to $T_{k}$ above. We do not observe values of r>T or values of v>T−r.

We assume that the camera recovery time *r* has a truncated gamma distribution

(6) $$f_{r}\left(r\right)\sim \text{Gamma}\left(r,\alpha ,\lambda \right),0<r\leq T$$

where *λ* > 0 is the rate and *α* > 0 is the shape parameter. The gamma distribution is suitable for modelling camera recovery times, as times are constrained to be positive, with most times clustering around the mean of the distribution, but with some shorter and some longer times. While the normal distribution also has this latter property, it does not constrain times to be positive, and unlike the gamma distribution, it does not have a shape parameter, and therefore it is less flexible.

For retrigger delay v, we assume an exponential distribution

(7) $$f_{v}\left(v\right)\sim \text{Exponential}\;\left(v,\mu \right),0<v\leq T-r$$

where *μ* > 0 is the rate parameter. The rate μ might be modelled as a function of distance from the camera and, where relevant, group size.

If the observed times truncated at *T* are $t_{i},i=1,\ldots ,n$, then the likelihood function is:

(8) $$L\left(\alpha ,\lambda ,\mu \right)=\prod_{i=1}^{n}f_{t}\left(t_{i}\right)$$

where $f_{t}\left(t\right)$ is the probability density function of t=r+v.

We can then estimate the mean time intervals between retriggers as the sum of camera recovery time r and retriggering time v as:

(9) $$\hat{E}\left(t\right)=\hat{E}\left(r\right)+\hat{E}\left(v\right)=\frac{\hat{\alpha }}{\hat{\lambda }}+\frac{1}{\hat{\mu }}$$

The full formulation is available in Appendix S1.

### 2.4 Implementation with real data

In our field study, we used the Cuddebback C2 (Green Bay, WI, United States). The camera manual suggested a recovery time of ca. 1 s. To assess the manufacturer‐specified time interval, we conducted an experiment, in which we tested 10 cameras for 2 min each by moving a hand up down directly in front of the PIR sensor and then calculated mean camera recovery time across and within the individual cameras.

One hundred eight of these camera traps were deployed in a year‐round survey (May 2018–August 2019) in the Bavarian Forest National Park and in a part of the neighbouring Šumava National Park (see Henrich et al., 2022 for details). The duration between triggers was set to the minimum (‘FAP’: fast as possible), and a series of five photographs was recorded each time the camera trap was triggered, which all get the same time stamp.

We used data on red deer (*Cervus elaphus*), roe deer (*Capreolus capreolus*) and wild boar (*Sus scrofa*) to estimate mean time intervals between triggers from pairs of consecutive images. Preliminary exploration of the data suggested that all species showed behavioural responses to camera traps, although to a different degree.

Three different (sub‐)sets of the data set were used: (1) the whole data set for which we assumed that all photographs with a time difference of less than 5 min to each other could be ascribed to the same group of animals, creating an ‘independent event’ (Henrich et al., 2022) (referred to as ‘full dataset’). We tested the influence of different time interval thresholds for the definition of an independent event on the resulting number of events and found stable results for thresholds between 5 and 60 min (Henrich et al., 2022, Figure S1). (2) Additionally, we randomly sampled 120 events with at least two photo series per species from the data set and randomly selected two consecutive photo series from within these events (referred to as ‘events checked dataset’). The first 100 sampled pairs of photo series of each species were manually checked to make sure that they showed the same individuals that did not leave the FOV between photo series with a high probability (based on body characteristics and the movement path of the animals across the FOV). For both red deer and roe deer, 15 events were excluded because these criteria were not met, while this was the case for 35 events in wild boar. These events were replaced by consecutive pairs of photo series from the remaining 20 events of each random sample. (3) The data set was further reduced to observations within a distance of 7.5 m, for which a sample size of 50 events was randomly selected from the appropriate subset of events for each species. A post was placed at a distance of 7.5 m from the camera trap at each camera trap location, allowing an easy assignment of animals to distances below or above that threshold (referred to as ‘within 7.5 m dataset’). The proposed statistical approach to derive the mean time interval between triggers was only applied to the full data set. Empirically derived estimates of the mean time interval between triggers were calculated for all three datasets.

We tested the influence of different truncation times *T* on the estimated mean time interval between triggers in the range of 15.5–40.5 s. To explore potentially more objective choices for selecting *T*, we also assessed two other truncation times when calculating *T* empirically. First, we selected *T* as the value corresponding to the third quartile of the data. Second, we set *T* to the value corresponding to 50% of the area under the curve of the histogram of time intervals between pairs of consecutive images. For doing this, we used the function ‘smooth. spline’ in R and set the smoothing parameter to 0.1. We then used the function ‘integrate’ to derive the value that corresponded to 50% of the area under the curve.

Last, we calculated the extent to which density *D* would be underestimated, when not accounting for reduced temporal survey effort due to longer‐than‐expected mean time intervals between triggers. Reference estimates for summer (June–August 2018), autumn (September–November 2018), winter (December 2018–February 2019) and spring (March–May 2019) were derived from a CTDS analysis applied to the full data set, using the first photograph of each photo series as a snapshot moment. For red deer and roe deer, they are equivalent to those presented in Henrich et al. (2022). For wild boar, the parameters were derived in the same way. We repeated the same analyses, but set $\hat{t}$ to 2, 6 and 9 s to represent the manufacturer specified recovery time, as well as the minimum time interval and the mean time interval between triggers as derived in the experimental setting. In addition, we filtered the data set to predefined snapshot intervals *t* = 2 s, *t* = 6 s and *t* = 9 s, decreasing *n*_*k*_ and affecting the estimate of the time spent active per day before recalculating the population density with CTDS to assess biases when using the snapshot approach.

We assumed a common camera recovery time model, and species‐specific retrigger delay models due to different movement behaviours among species. Assumed prior distributions are presented in Table 1. A Metropolis‐Hastings algorithm was used with 10,000 iterations, including a burn‐in period of 4000 iterations.

**TABLE 1:** Prior distributions for the shape parameter *α* and rate *λ* of the truncated Gamma distribution, and the species‐specific rates *μ*_1_, *μ*_2,_ *μ*_3_ (corresponding to red deer, roe deer and wild boar, respectively) for the exponential distribution.

Parameter	Prior distribution
α	lognormal(log(40), 1.0)
λ	lognormal(log(4.5), 1.0)
$\mu_{1}$	lognormal(log(0.4), 1.0)
$\mu_{2}$	lognormal(log(0.4), 1.0)
$\mu_{3}$	lognormal(log(0.4), 1.0)

## 3 RESULTS

When the time interval between photo series was tested experimentally, the mean was 8.82 s across 10 camera traps with a range between 6.3 and 43.6 s across cameras (Table S1).

**FIGURE 1:**
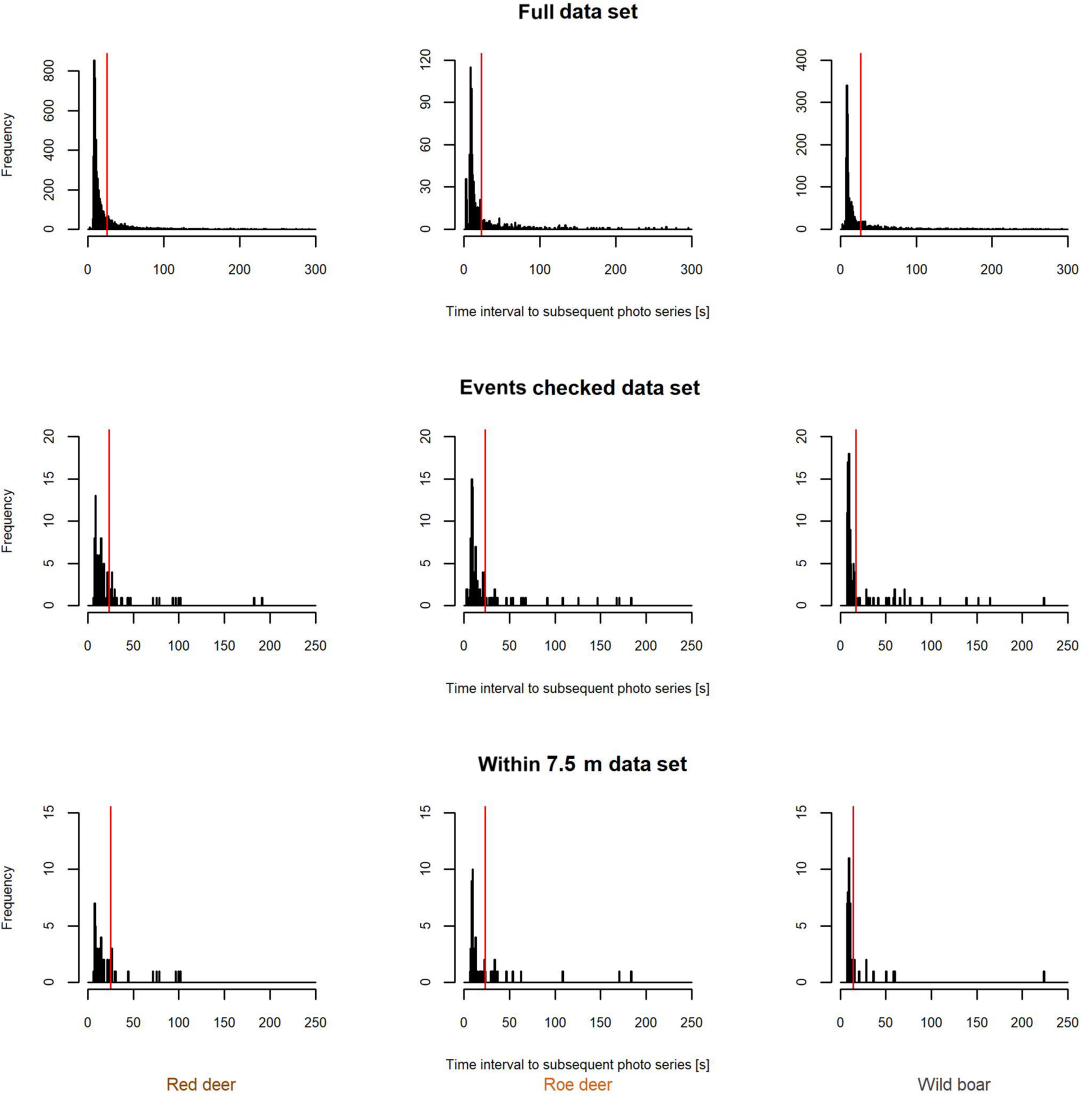
Time interval distributions between retriggers for the three species (red deer, roe deer, wild boar) for the full data sets (above), subsets (*n* = 100), including only intervals between pictures with the same animals (middle, *n* = 100) and subsets including additionally only those intervals for which animals were at short observation distances (bottom, *n* = 50). The vertical red line indicates the 3rd quartile of the time interval distribution data.

The sample sizes of time intervals between consecutive pictures were 2024, 5872 and 815 for wild boar, red deer and roe deer, respectively. The time interval distributions for all three species are similar with a peak around 10 s and a long tail (Figure 1). Subsets of the data (*n* = 100) including only intervals between pictures with the same animals (data set 2) show the same distribution pattern as the full data set (data set 1), but with a considerably reduced tail. When filtering for intervals between consecutive pictures with the same animals (*n* = 50) that are within 7.5 m to the camera (data set 3), the time interval distribution does not change.

**FIGURE 2:**
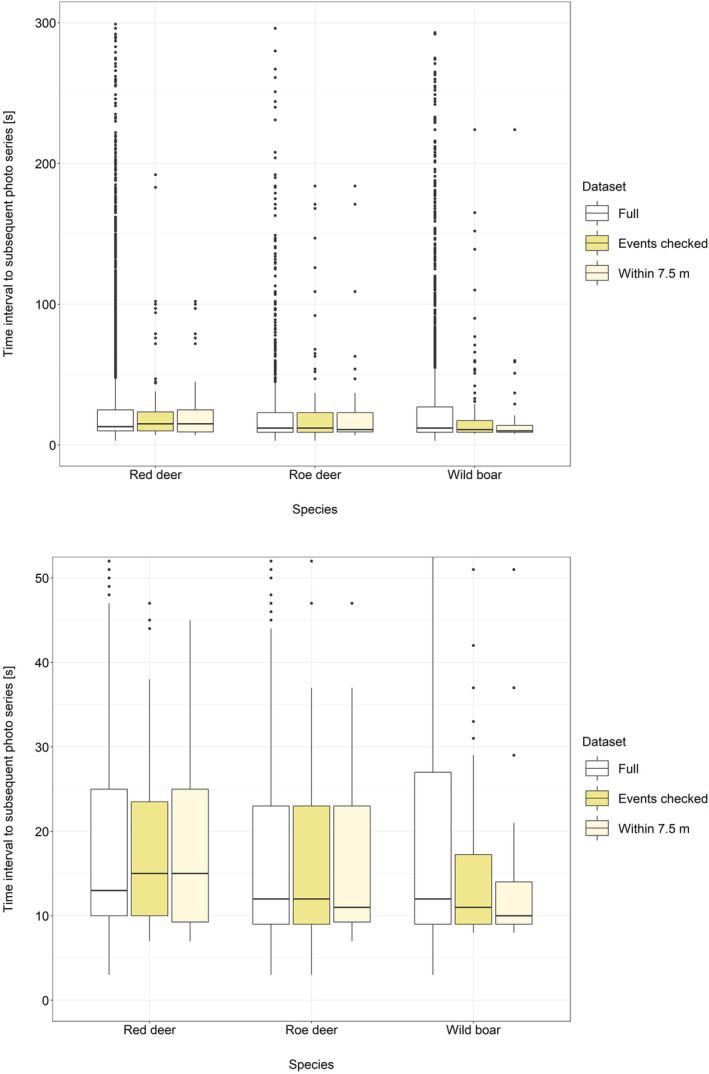
Comparison of mean time intervals between triggers for the three data sets and species with truncation time set to the 3rd quartile of the full data set (above: no truncation of datapoints; below: truncation of datapoints for better visibility of differences in mean time intervals between triggers).

Mean time intervals between triggers were similar regardless of whether they were estimated from the full or the reduced data sets, but differed with truncation time *T* (Table 2, Figure 2). Exemplified by setting *T* to the 3rd quartile of the time interval data, estimates for the different data sets were within less than 1.5 s for each species and data set, with the exception of the full data set for wild boar which differed by a maximum of 1.96 s relative to the other two data sets. Setting *T* to the value representing 50% of the area under the histogram curve (Figure S1) results were very similar with 13.21, 12.55 and 12.6 s for red deer, roe deer and wild boar, respectively.

**TABLE 2:** Mean time intervals between consecutive triggers for red deer, roe deer and wild boar for the three different data sets.

Species	Truncation time *T* [s]	Mean time intervals between triggers [s]		
		(1) Full data set	(2) Manually checked, same animals	(3) Manually checked, same animals and within 7.5 m
Red deer	3rd quartile	12.45 (4.36) [25 s]	12.96 (4.15) [23.5 s]	13.29 (5.12) [25 s]
	15.5	10.5 (2.07)	11.04 (2.51)	10.79 (2.69)
	20.5	11.56 (3.23)	12.17 (3.3)	11.76 (3.41)
	25.5	12.45 (4.36)	13.27 (4.53)	13.29 (5.12)
	30.5	13.32 (5.53)	14.5 (5.97)	14.67 (6.5)
	40.5	14.65 (7.5)	15.41 (7.21)	15.05 (6.89)
	None	26.74 (37.02)	24.87 (31.13)	24.36 (25.19)
Roe deer	3rd quartile	11.33 (4.74) [23 s]	11.6 (4.78) [23 s]	11.61 (4.02) [23 s]
	15.5	9.58 (2.93)	9.88 (2.7)	10.24 (1.87)
	20.5	10.47 (3.81)	10.39 (3.35)	10.97 (3.05)
	25.5	11.54 (4.99)	11.92 (5.14)	11.92 (4.44)
	30.5	12.15 (5.8)	12.55 (5.99)	12.38 (5.23)
	40.5	13.22 (7.47)	13.82 (7.79)	14.36 (8.1)
	None	27.32 (40.5)	25.48 (35.77)	25.2 (36.2)
Wild boar	3rd quartile	12.14 (4.81) [27 s]	10.72 (2.42) [17.25 s]	10.03 (1.62) [14 s]
	15.5	10.07 (2.18)	10.33 (1.99)	10.28 (1.92)
	20.5	11.05 (3.29)	10.82 (2.55)	10.55 (2.24)
	25.5	11.78 (4.29)	11.09 (3.04)	10.79 (2.73)
	30.5	12.64 (5.55)	11.54 (4.11)	11.6 (4.64)
	40.5	13.87 (7.45)	12.34 (5.81)	12.15 (5.92)
	None	30.13 (44.2)	25.68 (36.32)	19.06 (31.96)

*Note*: Standard deviations are shown in parentheses. The truncation time *T* of the respective data set, corresponding to the 3rd quartile of the raw time difference between consecutive photographs within the same event is shown in square brackets.

**FIGURE 3:**
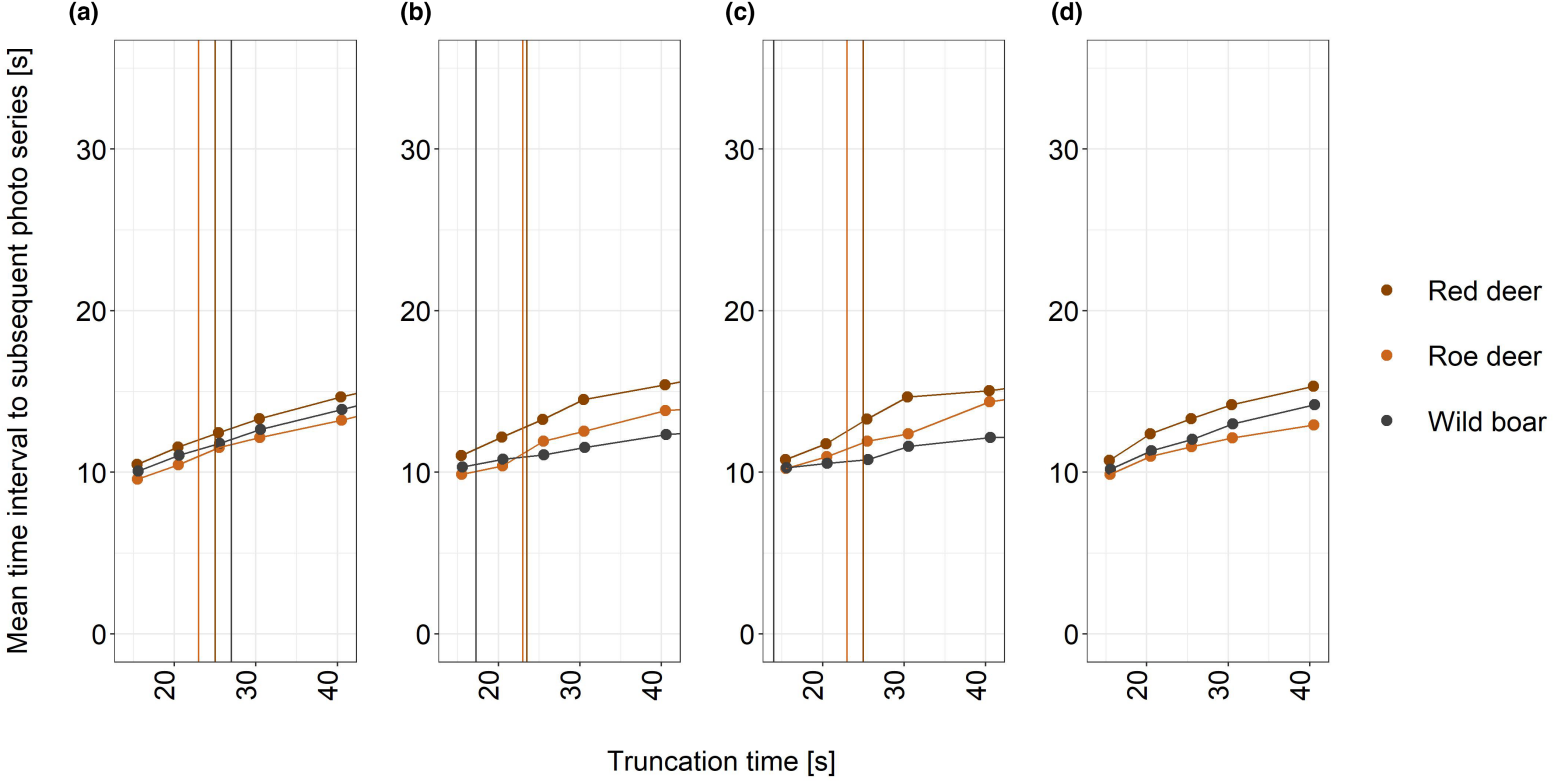
Mean time intervals between triggers as a function of truncation time for the three data sets (a—full, b—events checked, c—only distances up to 7.5 m) and the model‐based estimates (d). The vertical lines indicate the 3rd quartile of the time interval data for the three species.

When comparing the empirically derived mean time intervals between triggers with estimates based on the above formulated model for a range of truncation times *T* (15.5, 20.5, 25.5, 30.5 and 40.5 s) results were very similar between both approaches (Tables 2 and 3, Figure 3, trace plots in Figures S2–S7).

**TABLE 3:** Estimates of mean camera recovery time $\hat{E}\left(r\right)$, mean retrigger delay $\hat{E}\left(v\right)$ and mean interval $\hat{E}\left(t\right)$ (standard errors in parentheses) for truncation values of *T* = 15.5, 20.5, 25.5, 30.5 and 40.5 s. Also shown are 95% credible intervals for mean interval.

$\hat{E}\left(r\right)$	Species	$\hat{E}\left(v\right)$	$\hat{E}\left(t\right)$	95% CI
T=15.5				
9.56 (0.08)	Red deer	1.17 (0.10)	10.74 (0.06)	10.63, 10.85
	Roe deer	0.30 (0.08)	9.86 (0.09)	9.69, 10.04
	Wild boar	0.64 (0.08)	10.20 (0.07)	10.06, 10.34
T=20.5				
8.26 (0.09)	Red deer	4.12 (0.18)	12.38 (0.13)	12.15, 12.65
	Roe deer	2.73 (0.20)	10.99 (0.18)	10.65, 11.35
	Wild boar	3.08 (0.16)	11.33 (0.13)	11.09, 11.60
T=25.5				
7.94 (0.06)	Red deer	5.37 (0.16)	13.31 (0.14)	13.05, 13.58
	Roe deer	3.64 (0.22)	11.58 (0.21)	11.19, 12.00
	Wild boar	4.10 (0.15)	12.04 (0.14)	11.78, 12.32
T=30.5				
7.69 (0.06)	Red deer	6.50 (0.17)	14.19 (0.15)	13.91, 14.52
	Roe deer	4.43 (0.23)	12.12 (0.22)	11.70, 12.57
	Wild boar	5.31 (0.19)	13.00 (0.18)	12.67, 13.37
T=40.5				
7.46 (0.06)	Red deer	7.85 (0.17)	15.31 (0.15)	13.91, 14.52
	Roe deer	5.47 (0.27)	12.93 (0.26)	11.70, 12.57
	Wild boar	6.72 (0.19)	14.18 (0.19)	12.67, 13.37

However, estimation of *t* is sensitive to the choice of truncation time *T*. Estimates of mean camera recovery time *r* decrease as *T* increases (Table 3). Estimation of mean retrigger delay *v* once the camera has recovered is even more sensitive to the choice of *T* and increases as *T* increases. Overall, estimated *t* seems to be less sensitive to *T* than either estimated *r* or estimated *v*. Sensitivity is greatest for smaller choices of *T*.

The retrigger delay *v* differs among species and is estimated to be longest for red deer, followed by wild boar and roe deer (Table 3). This is similar, when comparing time intervals between consecutive pictures between red and roe deer within camera locations. Here, time intervals were longer for red deer in 65% of all cases, suggesting behavioural differences between the species (Figure S8).

Estimates of *t* seem to asymptote already at values of *T* below 50 s (Figure 3), reflecting the increasing gaps of time interval data between consecutive pictures with increasing values of *T* (Figure 1). However, with single, large values of time intervals between consecutive pictures, estimates of *t* then continue to increase with increasing values of *T* and only show clear asymptotic values of *t* at very large T (Figure 1, Figure S8).

**FIGURE 4:**
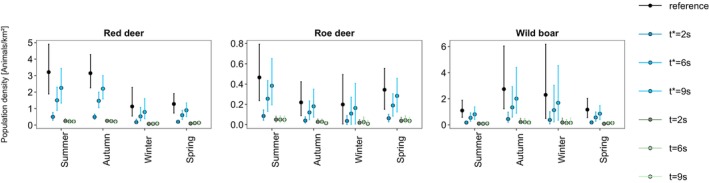
Population density estimates with CTDS for data sets of PIR sensor‐triggered photographs of red deer, roe deer and wild boar. The snapshot intervals *t* was calculated for the reference estimates by truncating the intervals between detections within independent events (consisting of photographs with a time difference in <5 min to each other) at the third quartile and computing the mean between successive triggers. In the other scenarios, the workflow was the same, but *t* was set to a different value (indicated in legend as t*), or the whole data set was subsampled to a predefined snapshot interval *t*. Error bars indicate the 95% confidence interval.

As density scales directly with the mean time interval between triggers, the correct representation of t has a major influence on the potential bias of estimates (Figure 4, Table S2). Using the experimentally derived mean across camera traps (rounded to 9 s) and lowest camera specific mean (6 s), population densities are underestimated by 18%–30% and 45%–53%, respectively.

This negative bias gets even stronger when analysing data with a snapshot approach, as a large proportion of data does not overlap with the predefined snapshot moments (Figure S9), leading to an underestimation of 87%–96% irrespective of the choice of *t* (Table S2).

## 4 DISCUSSION

Camera trap surveys with PIR motion sensor‐based recording of single pictures or short bursts require the correct estimation of mean time intervals between triggers. When animals are present in the detection zone, but are not recorded, effective survey duration is overestimated and estimation of density and abundance is downwardly biased. Clearly, this effect depends on the species studied, habitat, camera models and deployment method. Previous studies have suggested to derive the mean time interval between triggers from experimental testing of cameras, for example using minimum retrigger time. Our statistical approach offers a possibility to estimate mean time intervals between triggers from the time interval distribution of consecutive camera trap pictures. As observed in the field, estimated mean time intervals between triggers exceed the recovery time specified by the manufacturer and are also longer than experimentally derived intervals, since animals available for detection within the FOV do not constantly move and thus do not immediately retrigger the camera.

The proposed approach is more time‐effective, once programmed, compared with manual processing of data that requires filtering for a ‘clean’ subset containing only intervals between consecutive pictures with the same animal for deriving the mean time intervals between triggers. This is particularly the case in multi‐species surveys, when different species show different behaviours that cause the mean time interval between triggers to be longer than camera recovery time. The tracking of individuals across photo series and data processing would be very demanding. As density scales directly with *t* (Equations 2 and 4) in the CTDS model formulation, it is essential to derive effective survey duration with sufficient accuracy to avoid downwardly biased estimates of density. Thus, in CTDS surveys that use PIR motion sensor‐based recording of single pictures or short bursts, the parameter *t* originally defined as a predetermined snapshot interval, needs to be replaced by the estimated mean time interval between triggers.

### 4.1 Trade‐offs in defining truncation times

Estimation of the mean time interval between triggers is clearly sensitive to the choice of truncation time *T*. This is partly because observations of the same animal are increasingly mixed with observations of different animals when *T* gets larger. It is also possible that the same individual leaves and returns to a site, causing longer time intervals between subsequent triggers. In addition, an animal may be in the field of view, but shortly not visible, for example due to vegetation cover. This is indistinguishable from situations where animals remain stationary in the FOV and do not trigger the camera for a while. Whereas the latter contributes to the mean time intervals between triggers, the former does not.

For our cleaned data set (events checked data set), we had to remove 15 events for red and roe deer and 35 for wild boar, as they contained different individuals in consecutive picture series. This result suggests that truncation time *T* cannot extend over several minutes to avoid contamination of the time interval distribution between consecutive pictures. This will, however, certainly differ between species, their densities and habitats. If the chosen *T* is too large and includes a high proportion of time intervals between consecutive pictures with different individuals, the mean time interval between triggers will be biased upwards.

Although it would require additional cameras at a location to prove with certainty that an animal has left a spot and returned to it some time later, we found that leaving and returning likely contributes to longer estimated mean time intervals between triggers. By comparing pairs of consecutive images, we could not exclude the possibility that in 4%, 7% and 11% of image pairs red deer, roe deer and wild boar left and returned to the FOV within short time periods. Filtering those image pairs would lead to a reduction in estimated mean time intervals between triggers with the exception of wild boar (red deer: 12.02/12.96, roe deer: 10.42/11.06, wild boar: 11.6/10.42).

In our study, a *T* around 15 s seems to be insufficient to estimate the rate of the exponential distribution separately from fitting the gamma distribution, even though the precision of the model estimates is high. The trace plots improve as *T* increases, perhaps because of strong correlations between parameters when truncation is too severe (and *λ* are highly correlated, but no other correlations are close to one). To give more information for estimating the exponential rate, we need to take a larger value of *T*. However, the larger the value we choose, the greater the risk of contaminating the time interval distribution by including new animals or leaving and returning animals. This has the effect of widening the tail of the exponential distribution, which increases the estimated mean retrigger delay. The choice of *T* is therefore a compromise and requires careful consideration.

In principle, estimates of the mean time intervals between triggers must show asymptotic values with increasing *T*. This is also what we found in our study. However, as we likely did not have a ‘fully clean’ data set with only pairs of the individuals that did not leave the FOV, we consider the asymptotic values of *t* in this study as too large, and we offer suggestions for choosing *T* below.

### 4.2 Species‐specific behavioural differences

Some animal species may in general exhibit fewer micro‐movements that can potentially trigger a PIR sensor than others, for example short moments where an animal barely moves during foraging.

In some cases, animals may, however, also freeze in response to camera traps. In our data set, 19% of the roe deer and 34% of the red deer events included some form of behavioural reaction to the camera trap (Henrich et al., 2022), as well as 16% of wild boar events. Failure to account for behavioural responses to camera traps can strongly bias estimates of animal density, when they affect the staying time or position of animals in the FOV (Bessone et al., 2020; Houa et al., 2022). While behavioural responses can be corrected for when they can be classified as such (Delisle et al., 2023, submitted for publication), their effect on the retrigger delay v in data sets of PIR sensor‐triggered photographs cannot be directly observed. With our proposed approach to estimate *t*, the effect of species‐specific reactions to camera traps on this parameter can however be considered.

### 4.3 Practical considerations and implementation effort

Before a survey is started, a series of experiments can be conducted with the camera traps for evaluating the potential range of camera recovery times. Camera recovery times likely not only differ among camera models (often specified between 1 and 10 s, e.g. Palencia et al., 2022) but differ also considerably among cameras of the same type and even within the same camera over time. Using all or, if the number of cameras to be used is large, a subset of the cameras for assessing variation in recovery time before a survey can deliver important information. However, factors that potentially influence camera recovery time can be manifold, including ambient temperature, humidity, state of memory, writing speed on memory cards and energy supply. As these factors will change during the course of a survey, any exploration of and testing before a survey can not replace the correct estimation of survey specific mean time intervals between triggers across the used set of cameras and under the prevailing field conditions upon completion of the survey. Similarly, these experiments should include the testing of different sensitivity settings of the cameras and resulting impact on retrigger delays. Experimental testing can help finding a useful setting that avoids both excessive retriggering of cameras due to overly sensitive settings and insufficient retriggering due to sensitivity settings that lead to longer delays. In pilot studies prior to the start of a survey, it may also be tested, whether animal reactions towards camera traps may require including retrigger delay v in the calculation. Ideally, this is tested under field conditions, where some occlusion at larger distances may occur due to vegetation. Distance‐dependent retrigger times would require limiting estimation of the mean time intervals between triggers of the camera traps to short distances to avoid interference with reduced detection probability at large distances when estimating animal abundance (i.e. the estimation of detection probability as a function of distance). If it is clear, for example from prior surveys that there are no behavioural reactions of animals to cameras, retrigger delays from natural behaviour are negligible and experimentally derived camera recovery time shows little variation under different conditions, it should be sufficient to just use the experimentally derived value for *t*.

While we consider it as appropriate to model the mean time interval between triggers with a common camera recovery time when estimating overall density and abundance for a survey, this may require a different approach when making local scale predictions for spatial models. If differences in camera recovery time are large, location‐specific estimates for camera recovery time may be needed to avoid biases in predictions.

After a survey has been completed, the time interval data should be manually inspected for each species. This is needed to select a suitable truncation time *T*, which may differ between species. For comparison with the statistical estimator of mean time intervals between triggers $\hat{E}\left(t\right)$, a subset of the time interval data between consecutive recordings should be filtered for only those pictures belonging to the same animal to get a ‘clean’ time interval distribution that is not contaminated with time intervals between pictures belonging to different animals. This subset can be further filtered to a subset with only short camera–animal observation distances, as we did in our study.

This data filtering will help to identify a meaningful truncation time *T* that largely excludes time intervals between consecutive pictures showing different animals but keeps time intervals between consecutive pictures of the same individuals that stay in the detection zone. Even for wild boar, where consecutive photographs were most frequently from different individuals because of their larger group sizes, the differences between the data sets were small in our case study. The careful exploration of the frequency distribution will further aid in the identification of a useful truncation time *T*. Furthermore, the repeated estimation of mean time intervals between triggers $\hat{E}\left(t\right)$ using different values for *T* will also help to assess whether and when $\hat{E}\left(t\right)$ starts to asymptote.

The use of the 3rd quartile of the time interval distribution between consecutive pictures or the value at 50% of the area under the curve, as done in our study, requires further investigation. Our justification for use was solely based on inspection of the frequency distribution of interval data between consecutive pictures (see Section 4.5).

The effort to implement the proposed method and to calculate the mean time interval between retriggers is minimal. Extracting the dates and times from camera trap still images can be done automatically. Running the code provided with this study will take a few minutes to hours per species, including calculation of variances, depending on sample sizes and computational resources. The required manual effort is only a small fraction compared to other elements of the workflow to estimate population density of unmarked species from camera trapping data.

### 4.4 Validation of population density estimates

In our field study, true population density is unknown as free‐living deer cannot be counted directly and the rates of births, deaths, immigration and emigration cannot be quantified easily. However, estimates of the summer densities of red deer could be directly compared with an independent estimate obtained by spatially explicit capture–recapture (SECR) analyses, based on the genotyping of faeces sampled in the same area. Using a mean time interval between triggers based on setting *T* to the third quartile resulted in CTDS estimates that were very similar to the SECR estimates, with a high overlap of the 95% confidence intervals (Tourani et al., 2023). The same was true for REM estimates obtained with the same data set and GPS telemetry‐derived movement speed estimates (Henrich et al., 2022).

### 4.5 Recommendations for future research

The formal approach we presented here is one way towards including non‐observation times routinely into estimating animal abundance. We recommend that users of CTDS with trigger‐based recording of images include the proposed approach into their estimation of animal abundance, if it cannot be excluded that animal behaviour leads to retrigger delays. It should be tested how much animal abundance estimates change by including our approach for deriving values for *t*. It would be very useful to validate CTDS with trigger‐based recording of images in populations of known sizes using the proposed approach. This would help to better understand the magnitude of the effect of non‐observation times and the usefulness of the suggested approach compared to, for example experimental testing of camera recovery time.

The absence of an objective criterion for selecting *T* leaves also potential for future research. It would be important to better understand how longer time intervals between triggers are generated. It is possible that time intervals, say longer than 40–50 s, are primarily not caused by non‐moving animals, but by the same animals leaving and returning to the camera site. Consequently, these time intervals are not relevant for calculating mean time intervals between triggers and finding a solution to discriminate between the two would be very useful. This could be studied by installing more than one camera at a location to observe animal behaviour and movement from different angles. This may also help to understand when estimated mean time intervals between triggers asymptote as a function of *T*. Furthermore, the use of AI approaches to remove pairs of images with different individuals would help to pre‐filter or classify data and to make more informed selections of *T* and thus obtain more accurate estimates of *t*.

It could also be assessed whether fixing camera recovery time to the value derived from an experimental setup and only estimating v would help in deriving mean time intervals between triggers. Similarly, it would be interesting to assess whetherv is distance dependent and animals at larger distances are more likely to go undetected because stronger movements are needed to trigger the camera traps. Even if technological advancements of camera traps potentially reduce the camera recovery time *r* to a negligible duration, the issue of retrigger delay *v*, affected by non‐moving animals within the FOV, will remain and will need to be considered.

## 5 CONCLUSION

Camera trap surveys relying on PIR motion sensor‐based recording of pictures have to deal with non‐observation times caused by camera recovery times and possibly also retrigger delays due to non‐moving animals. The estimation of effective survey duration is critical to avoid underestimation of animal density. The suggested approach helps estimating mean time intervals between triggers without time‐consuming manual processing of pictures. Our findings show that estimated mean time intervals between triggers are very similar to empirically derived estimates based on manually filtered data sets. Nevertheless, the suggested approach still has limitations, as the estimation of the mean time interval between triggers is sensitive to the choice of the truncation time of interval data. However, the advantages of accounting for non‐observation times will likely offset potential inaccuracies in the estimator for reducing underestimation of animal abundance, and future development should lead to an improved performance of the estimator.

## AUTHOR CONTRIBUTIONS

**Hjalmar S. Kühl:** Conceptualization (lead); project administration (lead); supervision (lead); writing – original draft (lead); writing – review and editing (lead). **Stephen T. Buckland:** Conceptualization (equal); formal analysis (equal); methodology (lead); software (lead); visualization (supporting); writing – review and editing (supporting). **Maik Henrich:** Conceptualization (supporting); Data curation (lead); formal analysis (supporting); visualization (lead); writing – review and editing (supporting). **Eric Howe:** Conceptualization (supporting); methodology (supporting); writing – review and editing (supporting). **Marco Heurich:** Funding acquisition (supporting); supervision (equal); writing – review and editing (supporting).

## CONFLICT OF INTEREST STATEMENT

The authors declare no conflict of interest.

## Supporting information


Appendix S1

Data S1

## Data Availability

The time interval data for red deer, roe deer and wild boar, the R‐script for estimating mean time intervals between triggers and the experimentally derived camera recovery times are available as supplementary files (Data S1).
