# Supplementary material for: Estimating effective survey duration in camera trap distance sampling surveys
Source: Ecol Evol. 2023 Oct 13;13(10):e10599. doi: 10.1002/ece3.10599 (PMC10571013; doi:10.1002/ece3.10599)
Supplement: Supplementary file 1 — Appendix S1 [file ECE3-13-e10599-s002.docx]

Supporting Information

**Methods**

We assume that the recovery time *r* has a truncated gamma distribution with probability density function (pdf):

$$f_{r}\left( r \right)=\frac{\lambda^{\alpha}r^{\alpha-1}exp(-\lambda r)}{c(r,\alpha,\lambda)\Gamma(\alpha)} , 0<r\leq T$$

where $c(r,\alpha,\lambda)$ ensures that the pdf integrates to 1:

$$c(r,\alpha,\lambda)=\int_{0}^{T} \frac{\lambda^{\alpha}r^{\alpha-1}exp(-\lambda r)}{\Gamma(\alpha)}dr=\frac{\lambda^{\alpha}\int_{0}^{T} r^{\alpha-1}exp(-\lambda r)}{\Gamma(\alpha)} dr=\frac{\gamma(\alpha,\lambda T)}{\Gamma(\alpha)}$$

where $\gamma(\alpha,\lambda r)$ is the incomplete gamma function.

Thus

$$f_{r}\left( r \right)=\frac{\lambda^{\alpha}r^{\alpha-1}exp(-\lambda r)}{\gamma(\alpha,\lambda T)} , 0<r\leq T$$

For re-triggering time *v*, we assume an exponential distribution with probability density function:

$$f_{v}\left( v \right)=\frac{\mu exp(-\mu v)}{b(v,\mu)} , 0<v\leq T-r$$

where $b(v,\mu)$ ensures that the pdf integrates to 1:

$$b(v,\mu)=\int_{0}^{T-r} \mu\exp\left( -\mu v \right)dv=1-exp[-\mu\left( T-r \right)]$$

Thus

$$f_{v}\left( v \right)=\frac{\mu exp(-\mu v)}{1-exp[-\mu\left( T-r \right)]} , 0<v\leq T-r$$

The rate $\mu$ might be modelled as a function of distance from the camera and, where relevant, group size.

If the observed times truncated at *T* are $t_{i} i=1,\ldots,n$ , then the likelihood function is:

$$L\left( \alpha,\lambda,\mu\right)=\prod_{i=1}^{n} f_{t}(t_{i})$$

where $f_{t}(t)$ is the probability density function of $t=r+v$.

The joint density of *r* and *v* is:

$$f_{r,v}\left( r,v \right)=\frac{\lambda^{\alpha}r^{\alpha-1}\mu exp[-\left( \lambda r+\mu v \right)]}{\gamma(\alpha,\lambda T)1-exp[-\mu\left( T-r \right)]} , 0<r\leq T, 0<v\leq r$$

If we define the transformation $u=r, t=r+v$, then the Jacobian of the transformation is 1, and so

$$f_{u,t}\left( u,t \right)=\frac{\lambda^{\alpha}u^{\alpha-1}\mu exp\{-[(\lambda-\mu)u+\mu t]\}}{\gamma\left( \alpha,\lambda T \right)\{1-\exp\left[ -\mu\left( T-u \right) \right]\}} , 0<t\leq T, 0<u\leq t$$

Integrating out *u* gives us the pdf we need:

$$f_{t}\left( t \right)=\frac{\lambda^{\alpha}\mu exp(-\mu t)}{\gamma\left( \alpha,\lambda T \right)}\int_{0}^{t} \frac{u^{\alpha-1}exp[-(\lambda-\mu)u]}{\{1-\exp\left[ -\mu\left( T-u \right) \right]\}}du , 0<t\leq T$$

We maximise the likelihood to obtain maximum likelihood estimates $\hat{\alpha}, \hat{\lambda} \mathrm{and} \hat{\mu}$ of the parameters, and estimate $E(r)$ by numerically evaluating the following integral:

$$\int_{0}^{T} rf_{r}\left( r \right)dr=\int_{0}^{T} r.\frac{\hat{\lambda}^{\hat{\alpha}}r^{\hat{\alpha}-1}exp(-\hat{\lambda}r)}{\gamma(\hat{\alpha},\hat{\lambda}T)} dr=\frac{\int_{0}^{T} r^{\hat{\alpha}}exp(-\hat{\lambda}r)dr}{\int_{0}^{T} r^{\hat{\alpha}-1}exp(-\hat{\lambda}r)dr}$$

If *T* is sufficiently large that $f_{r}(r)$ is effectively not truncated, then we can estimate the mean recovery time by

$$\hat{E}\left( r \right)=\frac{\hat{\alpha}}{\hat{\lambda}}$$

However, we need the mean value of time interval *t*. Moreover, we need to estimate the mean of untruncated intervals. We fit to truncated time intervals to avoid contamination of the time interval distribution by detections of different animals or animal groups arriving during the interval to trigger the camera. A far more direct approach to estimating mean time interval would be to examine images, and determine whether successive detections are of the same animal or animal group, and we then simply take the sample mean of such intervals. However, there are two difficulties with this. First, it may be difficult to determine whether successive detections are of the same animal or animal group, and second, it would be very time-consuming to view all images to record the observed time intervals. By contrast, our approach, once programmed, gives results very quickly. The untruncated estimate we need is

$$\hat{E}\left( t \right)=\hat{E}\left( r \right)+\hat{E}\left( v \right)=\frac{\hat{\alpha}}{\hat{\lambda}}+\frac{1}{\hat{\mu}}$$

For multi-species surveys, the camera recovery time is likely to be the same across species. It is possible that animal behaviour could affect camera recovery time, but we expect any such effect to be small. Thus, to improve precision, we might fit a common recovery time model across species, while letting the exponential rate for the re-triggering time to vary by species. In this case, we have as before,

$$f_{r}\left( r \right)=\frac{\lambda^{\alpha}r^{\alpha-1}exp(-\lambda r)}{\gamma(\alpha,\lambda T)} , 0<r\leq T$$

However, we now have

$$f_{s}\left( v_{s} \right)=\frac{\mu_{s}exp(-\mu_{s}v_{s})}{1-exp[-\mu_{s}\left( T-r \right)]} , 0<v_{s}\leq T-r, s=1,\ldots,S$$

where *S* is the number of species in the multi-species analysis.

Thus

$$L\left( \alpha,\lambda,\mu_{1},\ldots,\mu_{S} \right)=\prod_{s=1}^{S} \prod_{i=1}^{n_{s}} f_{s}(t_{si})$$

where $n_{s}$ is the number of intervals of less than *T* for species *s*, and

$$f_{s}\left( t_{s} \right)=\frac{\lambda^{\alpha}\mu_{s}exp(-\mu_{s}t_{s})}{\gamma\left( \alpha,\lambda T \right)}\int_{0}^{t_{s}} \frac{u^{\alpha-1}exp[-(\lambda-\mu_{s})u]}{\{1-\exp\left[ -\mu_{s}\left( T-u \right) \right]\}}du , 0<t_{s}\leq T, s=1,\ldots,S.$$

Then we maximise the likelihood, and estimate species-specific mean time interval by:

$$\hat{E}\left( t_{s} \right)=\hat{E}\left( r \right)+\hat{E}\left( v_{s} \right)=\frac{\hat{\alpha}}{\hat{\lambda}}+\frac{1}{\hat{\mu}_{s}} , s=1,\ldots,S.$$

Some datasets may provide little information to split the distributions of camera recovery time *r* and re-trigger time *v*, in which case precision may be poor. Thus, a Bayesian approach may give better results, as we can set informative priors on the parameters of $f_{r}\left( r \right)$, given available information on camera recovery time.

**Results**

Figure 1: The smoothed histogram curves of time intervals between pairs of consecutive images for the three species. The vertical line indicates the 50% value for the area under the curve.


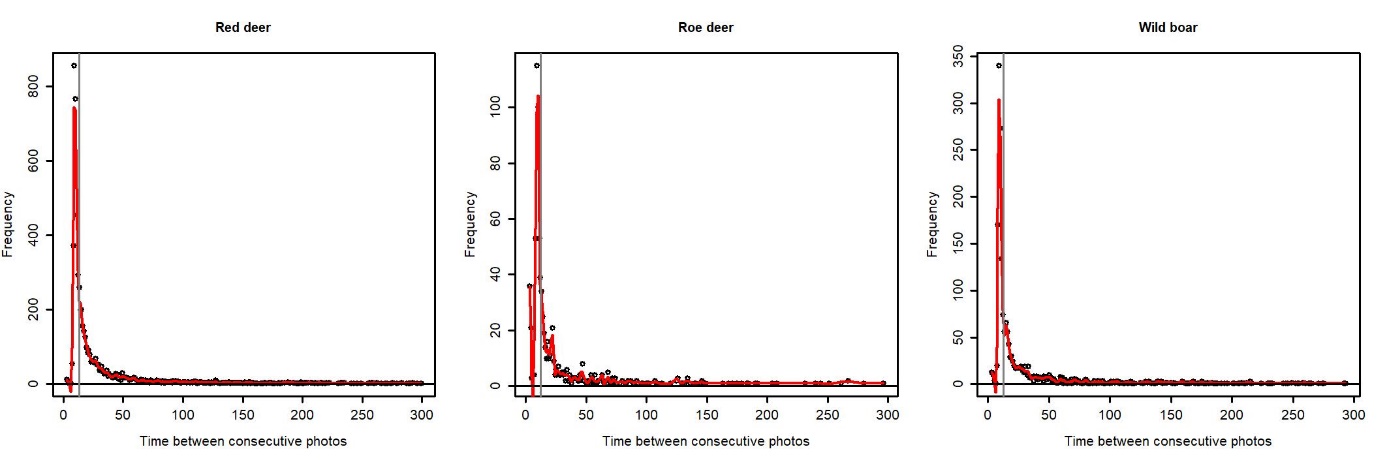


Figure 2: The trace plot corresponding to $T=15.5$ seconds. The first two plots refer to the estimation of the parameters α and λ of the Gamma distribution, the remaining three plots refer to the estimation of µ (mu) of the Exponential distribution for the three example species.

Figure 3: The trace plot corresponding to $T=20.5$ seconds. The first two plots refer to the estimation of the parameters α and λ of the Gamma distribution, the remaining three plots refer to the estimation of µ (mu) of the Exponential distribution for the three example species.

Figure 4: The trace plot corresponding to $T=25.5$ seconds. The first two plots refer to the estimation of the parameters α and λ of the Gamma distribution, the remaining three plots refer to the estimation of µ (mu) of the Exponential distribution for the three example species.

Figure 5: The trace plot corresponding to $T=30.5$ seconds. The first two plots refer to the estimation of the parameters α and λ of the Gamma distribution, the remaining three plots refer to the estimation of µ (mu) of the Exponential distribution for the three example species.

Figure 6: The trace plot corresponding to $T=40.5$ seconds. The first two plots refer to the estimation of the parameters α and λ of the Gamma distribution, the remaining three plots refer to the estimation of µ (mu) of the Exponential distribution for the three example species.

Figure 7: Mean time intervals between triggers as a function of truncation time for full dataset (A), event checked dataset (B), within 7.5m dataset (C), for the model-based estimates.


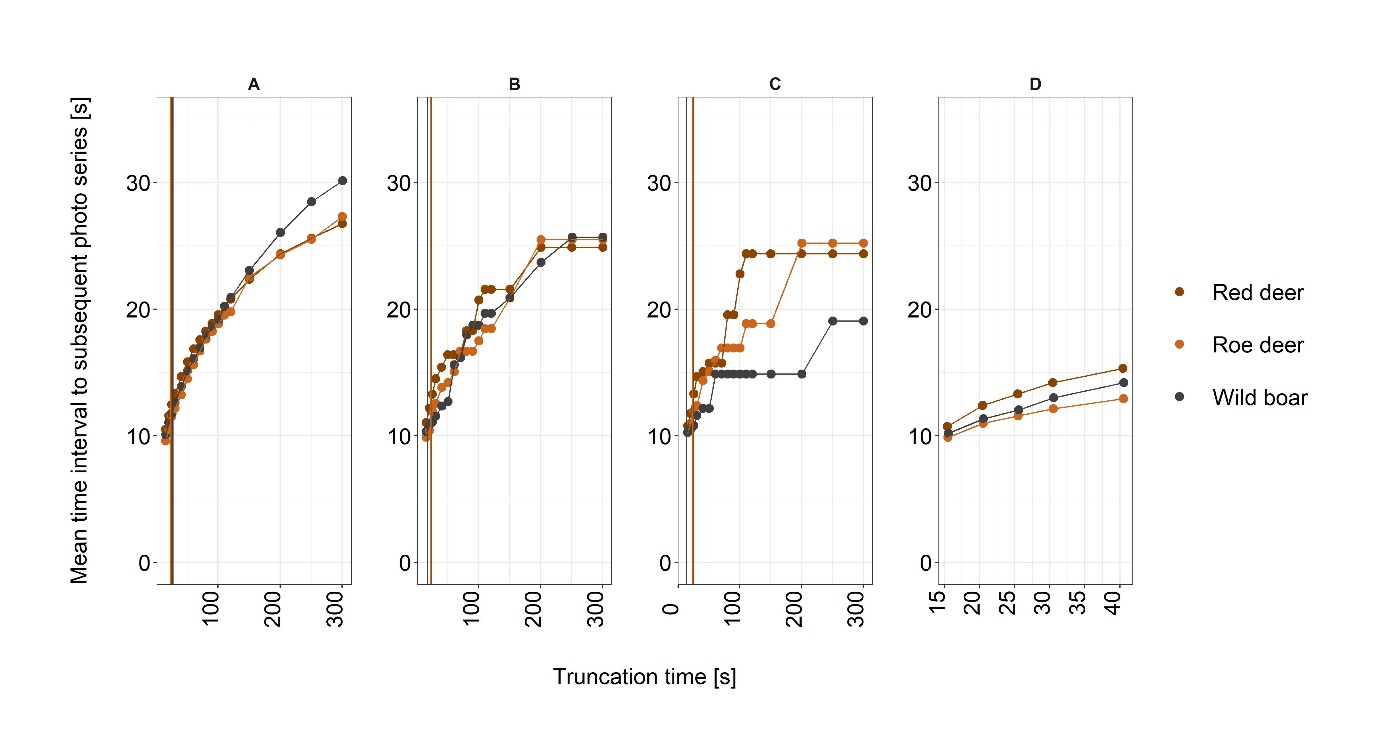


Figure 8: Differences in the time interval between consecutive pictures for the three species when not showing and showing behavioral reactions towards the camera traps.


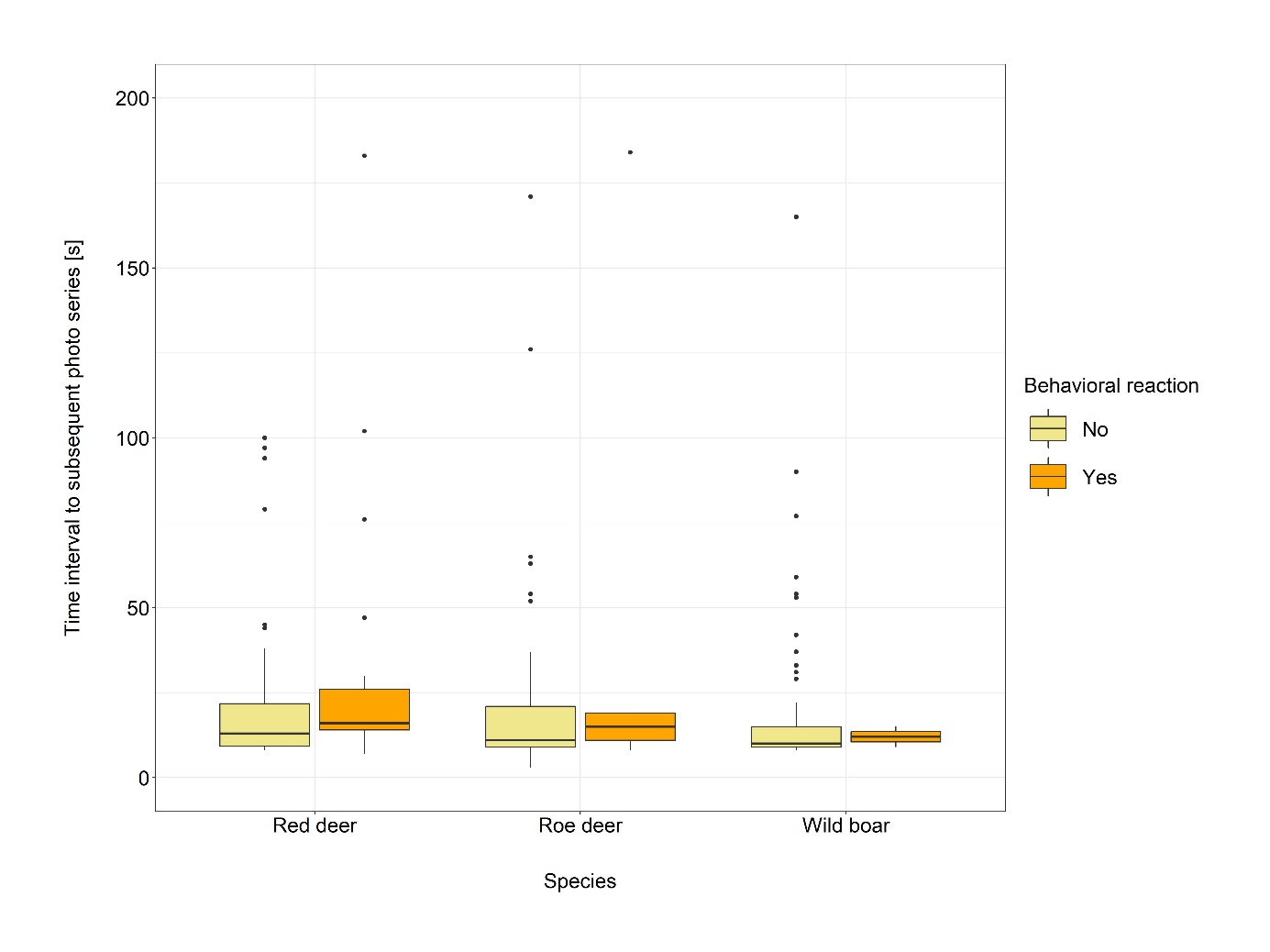


Figure 9: Effect of dataset reduction when applying a snapshot approach. As only some picture overlap with the predefined snapshot moments, the dataset gets smaller with larger values for *t*.


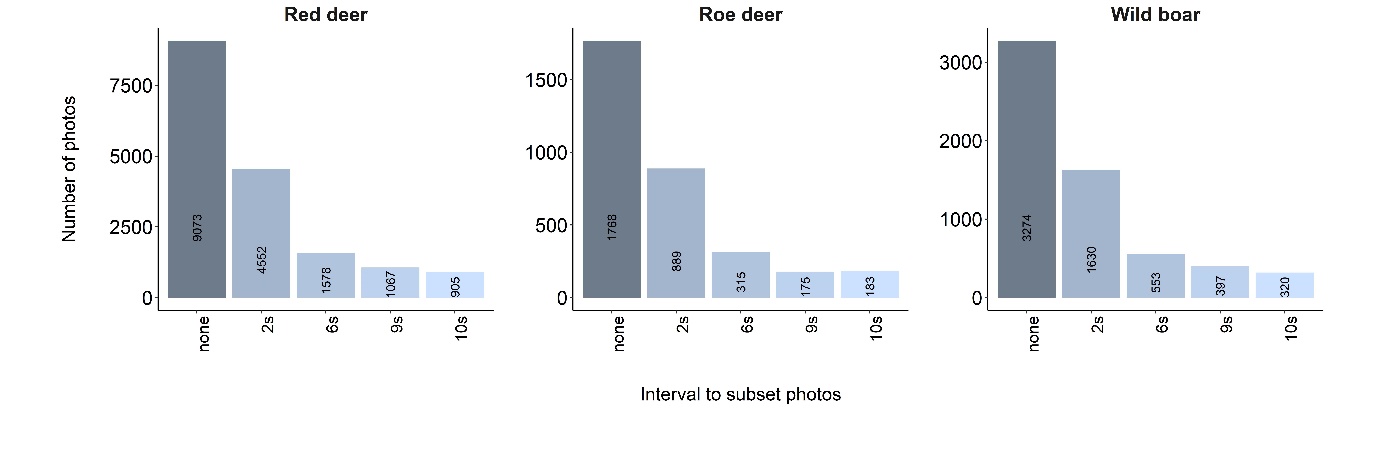


Supplementary Table 1: Results from experimental testing of camera traps to derive time intervals between triggers

| Camera | Min_interval | Median_interval | Mean_interval | n | weighted_av |
| --- | --- | --- | --- | --- | --- |
| 1 | 15 | 15 | 15,38 | 8 | 123 |
| 2 | 3 | 4 | 5,81 | 21 | 122 |
| 3 | 5 | 6 | 7,75 | 16 | 124 |
| 4 | 6 | 6,5 | 7,69 | 16 | 123 |
| 5 | 6 | 6 | 6,30 | 20 | 126 |
| 6 | 6 | 6 | 6,68 | 19 | 127 |
| 7 | 6 | 9,5 | 11,42 | 12 | 137 |
| 8 | 7 | 14,5 | 18,25 | 4 | 73 |
| 9 | 6 | 57 | 43,67 | 3 | 131 |
| 10 | 6 | 7 | 6,83 | 18 | 123 |
|  |  |  |  |  |  |
| Mean | 6,60 | 13,15 | 12,98 |  | 8,82 |
| Median | 6,00 | 6,50 | 7,69 |  |  |

Supplementary Table 2: Population density estimates with CTDS for datasets of PIR sensor-triggered photos of red deer, roe deer and wild boar. The snapshot intervals *t* were calculated for the reference estimates by truncating the intervals between detections within independent events (consisting of photos with a time difference of < 5 min to each other) at the third quartile and computing the mean between successive triggers. In the other scenarios, the workflow was the same, but *t* was set to a different value or the whole dataset was subsampled to a pre-defined snapshot interval *t*. Confidence intervals were computed by bootstrapping with 10000 iterations.

|  |  | Reference | | | | | | 2s | | | | | | | 2s predefined snaphots | | | | | | |
| --- | --- | --- | --- | --- | --- | --- | --- | --- | --- | --- | --- | --- | --- | --- | --- | --- | --- | --- | --- | --- | --- |
| Species | Season | Est. Dens. | | Lower 95% CI | Upper 95% CI | Individuals | Deployment days | Est. Dens. | | Lower 95% CI | Upper 95% CI | Individuals | Deployment days | Diff ref % | Est. Dens. | | Lower 95% CI | Upper 95% CI | Individuals | Deployment days | Diff ref % |
| Red deer | Summer | 3.21 | | 1.89 | 4.91 | 2419 | 9363 | 0.50 | | 0.30 | 0.76 | 2419 | 9363 | 84.40 | 0.25 | | 0.14 | 0.39 | 1177 | 9363 | 92.27 |
| Red deer | Autumn | 3.15 | | 2.26 | 4.28 | 2167 | 9404 | 0.49 | | 0.35 | 0.67 | 2167 | 9404 | 84.40 | 0.26 | | 0.19 | 0.35 | 1109 | 9404 | 91.75 |
| Red deer | Winter | 1.13 | | 0.55 | 2.29 | 234 | 3845 | 0.18 | | 0.09 | 0.36 | 234 | 3845 | 84.40 | 0.07 | | 0.04 | 0.15 | 117 | 3845 | 93.37 |
| Red deer | Spring | 1.28 | | 0.73 | 1.92 | 1217 | 6507 | 0.20 | | 0.11 | 0.30 | 1217 | 6507 | 84.40 | 0.09 | | 0.05 | 0.14 | 583 | 6507 | 92.63 |
| Roe deer | Summer | 0.47 | | 0.24 | 0.79 | 403 | 9363 | 0.09 | | 0.04 | 0.14 | 403 | 9363 | 81.69 | 0.05 | | 0.03 | 0.08 | 197 | 9363 | 89.14 |
| Roe deer | Autumn | 0.22 | | 0.09 | 0.42 | 229 | 9404 | 0.04 | | 0.02 | 0.08 | 229 | 9404 | 81.69 | 0.03 | | 0.01 | 0.06 | 130 | 9404 | 87.58 |
| Roe deer | Winter | 0.20 | | 0.01 | 0.49 | 41 | 3845 | 0.04 | | 0.00 | 0.09 | 41 | 3845 | 81.69 | 0.02 | | 0.00 | 0.05 | 22 | 3845 | 90.41 |
| Roe deer | Spring | 0.34 | | 0.15 | 0.55 | 248 | 6507 | 0.06 | | 0.03 | 0.10 | 248 | 6507 | 81.69 | 0.04 | | 0.02 | 0.07 | 101 | 6507 | 88.94 |
| Wild boar | Summer | 1.10 | | 0.58 | 1.90 | 712 | 9363 | 0.18 | | 0.09 | 0.31 | 712 | 9363 | 83.70 | 0.10 | | 0.05 | 0.17 | 354 | 9363 | 90.92 |
| Wild boar | Autumn | 2.74 | | 1.26 | 6.10 | 1992 | 9404 | 0.45 | | 0.20 | 0.99 | 1992 | 9404 | 83.70 | 0.22 | | 0.10 | 0.51 | 1020 | 9404 | 92.05 |
| Wild boar | Winter | 2.30 | | 0.46 | 6.02 | 888 | 3845 | 0.38 | | 0.07 | 0.99 | 888 | 3845 | 83.70 | 0.19 | | 0.03 | 0.51 | 439 | 3845 | 91.65 |
| Wild boar | Spring | 1.17 | | 0.53 | 2.08 | 829 | 6507 | 0.19 | | 0.09 | 0.34 | 829 | 6507 | 83.70 | 0.09 | | 0.05 | 0.17 | 419 | 6507 | 91.89 |
|  |  | Reference | | | | | | 6 s | | | | | | | 6s predefined snapshots | | | | | | |
| Species | Season | Est. Dens. | Lower 95% CI | | Upper 95% CI | Individuals | Deployment days | Est. Dens. | Lower 95% CI | | Upper 95% CI | Individuals | Deployment days | Diff ref % | Est. Dens. | Lower 95% CI | | Upper 95% CI | Individuals | Deployment days | Diff ref % |
| Red deer | Summer | 3.21 | 1.89 | | 4.91 | 2419 | 9363 | 1.50 | 0.89 | | 2.29 | 2419 | 9363 | 53.20 | 0.22 | 0.12 | | 0.36 | 400 | 9363 | 93.17 |
| Red deer | Autumn | 3.15 | 2.26 | | 4.28 | 2167 | 9404 | 1.47 | 1.06 | | 2.00 | 2167 | 9404 | 53.20 | 0.24 | 0.17 | | 0.32 | 370 | 9404 | 92.26 |
| Red deer | Winter | 1.13 | 0.55 | | 2.29 | 234 | 3845 | 0.53 | 0.26 | | 1.07 | 234 | 3845 | 53.20 | 0.08 | 0.03 | | 0.16 | 42 | 3845 | 92.83 |
| Red deer | Spring | 1.28 | 0.73 | | 1.92 | 1217 | 6507 | 0.60 | 0.34 | | 0.90 | 1217 | 6507 | 53.20 | 0.12 | 0.06 | | 0.19 | 206 | 6507 | 90.60 |
| Roe deer | Summer | 0.47 | 0.24 | | 0.79 | 403 | 9363 | 0.26 | 0.13 | | 0.43 | 403 | 9363 | 45.07 | 0.05 | 0.02 | | 0.08 | 75 | 9363 | 89.25 |
| Roe deer | Autumn | 0.22 | 0.09 | | 0.42 | 229 | 9404 | 0.12 | 0.05 | | 0.23 | 229 | 9404 | 45.07 | 0.03 | 0.01 | | 0.05 | 48 | 9404 | 87.11 |
| Roe deer | Winter | 0.20 | 0.01 | | 0.49 | 41 | 3845 | 0.11 | 0.00 | | 0.27 | 41 | 3845 | 45.07 | 0.03 | 0.00 | | 0.07 | 10 | 3845 | 86.87 |
| Roe deer | Spring | 0.34 | 0.15 | | 0.55 | 248 | 6507 | 0.19 | 0.08 | | 0.30 | 248 | 6507 | 45.07 | 0.04 | 0.02 | | 0.09 | 32 | 6507 | 87.34 |
| Wild boar | Summer | 1.10 | 0.58 | | 1.90 | 712 | 9363 | 0.54 | 0.28 | | 0.93 | 712 | 9363 | 51.10 | 0.09 | 0.05 | | 0.16 | 122 | 9363 | 91.87 |
| Wild boar | Autumn | 2.74 | 1.26 | | 6.10 | 1992 | 9404 | 1.34 | 0.61 | | 2.97 | 1992 | 9404 | 51.10 | 0.21 | 0.09 | | 0.48 | 350 | 9404 | 92.33 |
| Wild boar | Winter | 2.30 | 0.46 | | 6.02 | 888 | 3845 | 1.13 | 0.22 | | 2.96 | 888 | 3845 | 51.10 | 0.15 | 0.02 | | 0.42 | 116 | 3845 | 93.35 |
| Wild boar | Spring | 1.17 | 0.53 | | 2.08 | 829 | 6507 | 0.57 | 0.26 | | 1.02 | 829 | 6507 | 51.10 | 0.13 | 0.06 | | 0.27 | 161 | 6507 | 88.75 |
|  |  | Reference | | | | | | 9 s | | | | | | | 9s predefined snapshots | | | | | | |
| Species | Season | Est. Dens. | Lower 95% CI | | Upper 95% CI | Individuals | Deployment days | Est. Dens. | Lower 95% CI | | Upper 95% CI | Individuals | Deployment days | Diff ref % | Est. Dens. | Lower 95% CI | | Upper 95% CI | Individuals | Deployment days | Diff ref % |
| Red deer | Summer | 3.21 | 1.89 | | 4.91 | 2419 | 9363 | 2.26 | 1.33 | | 3.44 | 2419 | 9363 | 29.80 | 0.22 | 0.11 | | 0.34 | 269 | 9363 | 93.23 |
| Red deer | Autumn | 3.15 | 2.26 | | 4.28 | 2167 | 9404 | 2.21 | 1.59 | | 3.00 | 2167 | 9404 | 29.80 | 0.21 | 0.13 | | 0.27 | 226 | 9404 | 93.22 |
| Red deer | Winter | 1.13 | 0.55 | | 2.29 | 234 | 3845 | 0.79 | 0.38 | | 1.60 | 234 | 3845 | 29.80 | 0.09 | 0.03 | | 0.23 | 29 | 3845 | 91.72 |
| Red deer | Spring | 1.28 | 0.73 | | 1.92 | 1217 | 6507 | 0.90 | 0.51 | | 1.34 | 1217 | 6507 | 29.80 | 0.14 | 0.07 | | 0.22 | 173 | 6507 | 89.10 |
| Roe deer | Summer | 0.47 | 0.24 | | 0.79 | 403 | 9363 | 0.38 | 0.20 | | 0.65 | 403 | 9363 | 17.61 | 0.05 | 0.02 | | 0.09 | 49 | 9363 | 89.65 |
| Roe deer | Autumn | 0.22 | 0.09 | | 0.42 | 229 | 9404 | 0.18 | 0.07 | | 0.35 | 229 | 9404 | 17.61 | 0.02 | 0.00 | | 0.03 | 18 | 9404 | 93.07 |
| Roe deer | Winter | 0.20 | 0.01 | | 0.49 | 41 | 3845 | 0.16 | 0.01 | | 0.41 | 41 | 3845 | 17.61 | 0.01 | 0.00 | | 0.04 | 2 | 3845 | 95.61 |
| Roe deer | Spring | 0.34 | 0.15 | | 0.55 | 248 | 6507 | 0.28 | 0.13 | | 0.46 | 248 | 6507 | 17.61 | 0.04 | 0.01 | | 0.07 | 20 | 6507 | 89.08 |
| Wild boar | Summer | 1.10 | 0.58 | | 1.90 | 712 | 9363 | 0.81 | 0.42 | | 1.39 | 712 | 9363 | 26.64 | 0.11 | 0.05 | | 0.21 | 101 | 9363 | 90.07 |
| Wild boar | Autumn | 2.74 | 1.26 | | 6.10 | 1992 | 9404 | 2.01 | 0.92 | | 4.45 | 1992 | 9404 | 26.64 | 0.18 | 0.07 | | 0.37 | 204 | 9404 | 93.59 |
| Wild boar | Winter | 2.30 | 0.46 | | 6.02 | 888 | 3845 | 1.69 | 0.33 | | 4.43 | 888 | 3845 | 26.64 | 0.16 | 0.03 | | 0.52 | 71 | 3845 | 93.19 |
| Wild boar | Spring | 1.17 | 0.53 | | 2.08 | 829 | 6507 | 0.86 | 0.39 | | 1.53 | 829 | 6507 | 26.64 | 0.15 | 0.06 | | 0.29 | 129 | 6507 | 87.55 |
